# (5-Bromo-2-hydroxy­phen­yl)(phen­yl)methanone

**DOI:** 10.1107/S1600536808027578

**Published:** 2008-09-06

**Authors:** Feng-Ke Yang, Yi-Ning Ding, Wei Cheng, Ke Xu

**Affiliations:** a College of Chemical Engineering, Qingdao University of Science and Technology, Qingdao 266042, People’s Republic of China; b Key Laboratory of Advanced Materials, Qingdao University of Science and Technology, Qingdao 266042, People’s Republic of China

## Abstract

In the title compound, C_13_H_9_BrO_2_, the mol­ecular conformation is stabilized by an intra­molecular O—H⋯O hydrogen bond. In the crystal structure, weak inter­molecular C—H⋯O hydrogen-bonding inter­actions link the mol­ecules into chains along the *c*-axis direction.

## Related literature

For related literature, see: Dale *et al.* (1999 ▶); Sridhar & Saravanan (2001 ▶); Wiktor *et al.* (2000 ▶); Hester *et al.* (2001 ▶); Idrees *et al.* (2001 ▶); Zhou (2006 ▶).
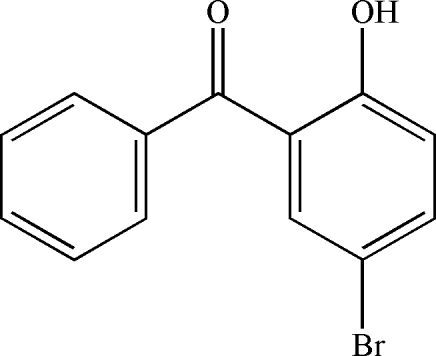


## Experimental

### 

#### Crystal data

C_13_H_9_BrO_2_
*M* *_r_* = 277.10
Monoclinic, 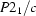

*a* = 15.938 (3) Å
*b* = 5.8929 (12) Å
*c* = 12.111 (2) Å
β = 106.15 (3)°
*V* = 1092.6 (4) Å^3^
*Z* = 4
Mo *K*α radiation
μ = 3.74 mm^−1^
*T* = 295 (2) K
0.30 × 0.20 × 0.10 mm

#### Data collection

Bruker SMART 1K CCD area-detector diffractometer
Absorption correction: multi-scan (*SADABS*; Sheldrick, 2004 ▶) *T*_min_ = 0.417, *T*_max_ = 0.689
4878 measured reflections
2292 independent reflections
1767 reflections with *I* > 2σ(*I*)
*R*_int_ = 0.026

#### Refinement

*R*[*F* ^2^ > 2σ(*F* ^2^)] = 0.035
*wR*(*F* ^2^) = 0.101
*S* = 1.08
2292 reflections
145 parameters
H-atom parameters constrained
Δρ_max_ = 0.36 e Å^−3^
Δρ_min_ = −0.61 e Å^−3^

### 

Data collection: *SMART* (Bruker, 2001 ▶); cell refinement: *SAINT* (Bruker, 2001 ▶); data reduction: *SAINT*; program(s) used to solve structure: *SHELXTL* (Sheldrick, 2008 ▶); program(s) used to refine structure: *SHELXTL*; molecular graphics: *SHELXTL*; software used to prepare material for publication: *SHELXTL* and local programs.

## Supplementary Material

Crystal structure: contains datablocks global, I. DOI: 10.1107/S1600536808027578/at2622sup1.cif

Structure factors: contains datablocks I. DOI: 10.1107/S1600536808027578/at2622Isup2.hkl

Additional supplementary materials:  crystallographic information; 3D view; checkCIF report

## Figures and Tables

**Table 1:** Hydrogen-bond geometry (Å, °)

*D*—H⋯*A*	*D*—H	H⋯*A*	*D*⋯*A*	*D*—H⋯*A*
O2—H2*A*⋯O1	0.82	1.85	2.570 (3)	146
C13—H13*A*⋯O2^i^	0.93	2.59	3.475 (3)	160

Symmetry code: (i) 
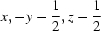
.

**Fig. 1.**
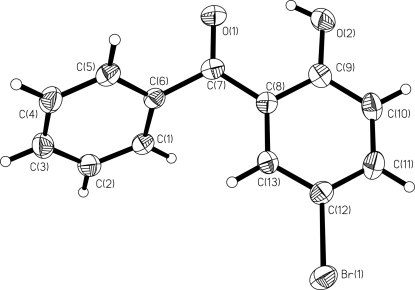
The structure of the title compound showing 30% probability displacement ellipsoids and the atom-numbering scheme.

**Fig. 2.**
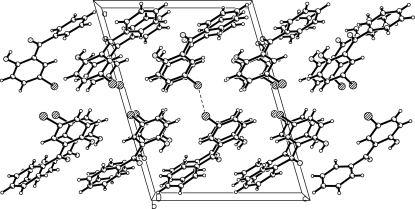
The packing of (I), viewed down the b axis.

## supplementary crystallographic information

### Comment

Monocondensed Schiff bases are attractive because of their intermediates in the synthesis of unsymmetrical multidentate Schiff base ligands and metal complexes, which serve as potential chelating agents and catalyst in synthesis and pharmaceutical fields (Hester *et al.*, 2001). New examples are being tested for their antitumor, (Idrees *et al.*, 2001). antimicroial and antiviral activities (Sridhar & Saravanan, 2001). We describe the structure of the title compound is a precursor of monocondensed Schiff bases.

In the title compound, bond lengths are slightly different from those in similar compounds. The C—Br bond length [1.896 (3) Å] is longer than others reported [1.865 (1) (Dale *et al.*, 1999) and 1.884 (2)Å (Wiktor *et al.*, 2000)]. Molecular conformation is stabilized by an intramolecular O—H···O hydrogen bond. In the crystal structure, weak intermolecular C—H···O hydrogen bonding interactions (Table 1) link the molecules into chains along the b-direction.

### Experimental

5-Bromo-2-hydroxybenzophenone was prepared *via* the Fries rearrangement of *p*-bromophenyl benzoate at 433 K with AlCl_3_ as the catalyst. The title compound was collected and washed with 10% diluted hydrochloric acid. Single crystals suitable for X-ray measurements were obtained by recrystallization from absolute ethanol and acetic ether (1:1,*v*/*v*) at room temperature.

### Refinement

All H atoms were placed at calculated positions and allowed to ride on their attached atoms, with C—H distance = 0.93 Å and O—H = 0.82 Å, and with *U*_iso_ =1.2 *U*_eq_ (C) and *U*_iso_ =1.5 *U*_eq_ (O).

### Crystal data

**Table:**

C_13_H_9_BrO_2_	*F*(000) = 552
*M**_r_* = 277.10	*D*_x_ = 1.685 Mg m^−^^3^
Monoclinic, *P*2_1_/*c*	Mo *K*α radiation, λ = 0.71073 Å
Hall symbol: -P 2ybc	Cell parameters from 1025 reflections
*a* = 15.938 (3) Å	θ = 1.3–27.0°
*b* = 5.8929 (12) Å	µ = 3.74 mm^−^^1^
*c* = 12.111 (2) Å	*T* = 295 K
β = 106.15 (3)°	Block, yellow
*V* = 1092.6 (4) Å^3^	0.30 × 0.20 × 0.10 mm
*Z* = 4	

### Data collection

**Table:**

Bruker SMART 1K CCD area-detector diffractometer	2292 independent reflections
Radiation source: fine-focus sealed tube	1767 reflections with *I* > 2σ(*I*)
graphite	*R*_int_ = 0.026
Thin–slice ω scans	θ_max_ = 26.7°, θ_min_ = 1.3°
Absorption correction: multi-scan (SADABS; Sheldrick, 2004)	*h* = −19→19
*T*_min_ = 0.417, *T*_max_ = 0.689	*k* = −7→6
4878 measured reflections	*l* = −14→15

### Refinement

**Table:**

Refinement on *F*^2^	Primary atom site location: structure-invariant direct methods
Least-squares matrix: full	Secondary atom site location: difference Fourier map
*R*[*F*^2^ > 2σ(*F*^2^)] = 0.035	Hydrogen site location: inferred from neighbouring sites
*wR*(*F*^2^) = 0.101	H-atom parameters constrained
*S* = 1.08	*w* = 1/[σ^2^(*F*_o_^2^) + (0.0519*P*)^2^ + 0.0875*P*] where *P* = (*F*_o_^2^ + 2*F*_c_^2^)/3
2292 reflections	(Δ/σ)_max_ < 0.001
145 parameters	Δρ_max_ = 0.36 e Å^−^^3^
0 restraints	Δρ_min_ = −0.61 e Å^−^^3^

### Special details

**Table:**

Geometry. All e.s.d.'s (except the e.s.d. in the dihedral angle between two l.s. planes) are estimated using the full covariance matrix. The cell e.s.d.'s are taken into account individually in the estimation of e.s.d.'s in distances, angles and torsion angles; correlations between e.s.d.'s in cell parameters are only used when they are defined by crystal symmetry. An approximate (isotropic) treatment of cell e.s.d.'s is used for estimating e.s.d.'s involving l.s. planes.
Refinement. Refinement of *F*^2^ against ALL reflections. The weighted *R*-factor *wR* and goodness of fit *S* are based on *F*^2^, conventional *R*-factors *R* are based on *F*, with *F* set to zero for negative *F*^2^. The threshold expression of *F*^2^ > σ(*F*^2^) is used only for calculating *R*-factors(gt) *etc*. and is not relevant to the choice of reflections for refinement. *R*-factors based on *F*^2^ are statistically about twice as large as those based on *F*, and *R*- factors based on ALL data will be even larger.

### Fractional atomic coordinates and isotropic or equivalent isotropic displacement parameters (Å^2^)

**Table:**

	*x*	*y*	*z*	*U*_iso_*/*U*_eq_	
Br1	0.42247 (2)	0.27571 (6)	0.49704 (3)	0.06162 (16)	
O1	0.18993 (15)	−0.6147 (4)	0.49901 (19)	0.0609 (6)	
O2	0.28487 (15)	−0.5189 (4)	0.70244 (19)	0.0613 (6)	
H2A	0.2498	−0.5930	0.6532	0.092*	
C1	0.11815 (19)	−0.1537 (5)	0.3198 (3)	0.0457 (7)	
H1A	0.1235	−0.0469	0.3779	0.055*	
C2	0.06832 (19)	−0.1057 (5)	0.2101 (3)	0.0518 (7)	
H2B	0.0378	0.0305	0.1950	0.062*	
C3	0.0635 (2)	−0.2590 (5)	0.1225 (3)	0.0580 (9)	
H3A	0.0308	−0.2246	0.0480	0.070*	
C4	0.1071 (2)	−0.4629 (5)	0.1454 (3)	0.0560 (8)	
H4A	0.1049	−0.5649	0.0861	0.067*	
C5	0.1541 (2)	−0.5160 (5)	0.2561 (3)	0.0484 (7)	
H5A	0.1816	−0.6563	0.2718	0.058*	
C6	0.16053 (17)	−0.3611 (5)	0.3442 (2)	0.0398 (6)	
C7	0.20826 (18)	−0.4302 (5)	0.4640 (3)	0.0432 (6)	
C8	0.27492 (18)	−0.2824 (4)	0.5359 (3)	0.0387 (6)	
C9	0.30999 (19)	−0.3350 (5)	0.6535 (3)	0.0469 (7)	
C10	0.3733 (2)	−0.1974 (6)	0.7230 (3)	0.0554 (8)	
H10A	0.3943	−0.2292	0.8011	0.066*	
C11	0.4053 (2)	−0.0134 (6)	0.6774 (3)	0.0564 (8)	
H11A	0.4476	0.0795	0.7245	0.068*	
C12	0.37446 (18)	0.0319 (5)	0.5617 (3)	0.0453 (7)	
C13	0.30905 (17)	−0.0964 (5)	0.4910 (2)	0.0408 (6)	
H13A	0.2876	−0.0595	0.4136	0.049*	

### Atomic displacement parameters (Å^2^)

**Table:**

	*U*^11^	*U*^22^	*U*^33^	*U*^12^	*U*^13^	*U*^23^
Br1	0.0542 (2)	0.0546 (2)	0.0743 (3)	−0.01334 (14)	0.01486 (18)	0.00225 (16)
O1	0.0721 (15)	0.0486 (12)	0.0597 (14)	−0.0165 (11)	0.0143 (12)	0.0081 (10)
O2	0.0680 (15)	0.0658 (14)	0.0489 (13)	−0.0075 (11)	0.0140 (11)	0.0156 (11)
C1	0.0468 (16)	0.0418 (14)	0.0496 (18)	−0.0017 (13)	0.0151 (14)	−0.0040 (13)
C2	0.0443 (17)	0.0504 (17)	0.057 (2)	0.0004 (13)	0.0087 (14)	0.0051 (15)
C3	0.0526 (19)	0.072 (2)	0.0452 (18)	−0.0139 (16)	0.0063 (15)	0.0036 (16)
C4	0.065 (2)	0.0589 (19)	0.0454 (18)	−0.0105 (16)	0.0173 (16)	−0.0108 (15)
C5	0.0524 (18)	0.0421 (15)	0.0526 (18)	−0.0039 (13)	0.0174 (15)	−0.0071 (13)
C6	0.0377 (14)	0.0398 (14)	0.0428 (16)	−0.0044 (11)	0.0128 (12)	−0.0016 (12)
C7	0.0458 (16)	0.0391 (14)	0.0484 (17)	−0.0014 (12)	0.0194 (13)	−0.0020 (12)
C8	0.0360 (14)	0.0399 (14)	0.0414 (16)	0.0049 (11)	0.0126 (12)	−0.0018 (11)
C9	0.0442 (16)	0.0540 (16)	0.0450 (18)	0.0055 (13)	0.0165 (13)	0.0029 (13)
C10	0.0492 (18)	0.077 (2)	0.0365 (17)	0.0005 (16)	0.0067 (14)	0.0010 (15)
C11	0.0470 (18)	0.072 (2)	0.0476 (19)	−0.0087 (15)	0.0084 (14)	−0.0105 (16)
C12	0.0402 (15)	0.0461 (15)	0.0516 (18)	−0.0020 (12)	0.0160 (13)	−0.0031 (13)
C13	0.0392 (15)	0.0443 (15)	0.0387 (16)	0.0046 (11)	0.0105 (12)	−0.0001 (12)

### Geometric parameters (Å, °)

**Table:**

Br1—C12	1.896 (3)	C5—C6	1.386 (4)
O1—C7	1.231 (3)	C5—H5A	0.9300
O2—C9	1.348 (4)	C6—C7	1.495 (4)
O2—H2A	0.8200	C7—C8	1.460 (4)
C1—C2	1.374 (4)	C8—C13	1.400 (4)
C1—C6	1.388 (4)	C8—C9	1.412 (4)
C1—H1A	0.9300	C9—C10	1.383 (4)
C2—C3	1.379 (5)	C10—C11	1.378 (4)
C2—H2B	0.9300	C10—H10A	0.9300
C3—C4	1.378 (4)	C11—C12	1.377 (4)
C3—H3A	0.9300	C11—H11A	0.9300
C4—C5	1.377 (4)	C12—C13	1.376 (4)
C4—H4A	0.9300	C13—H13A	0.9300
			
C9—O2—H2A	109.5	O1—C7—C6	118.0 (3)
C2—C1—C6	120.2 (3)	C8—C7—C6	120.4 (2)
C2—C1—H1A	119.9	C13—C8—C9	118.5 (3)
C6—C1—H1A	119.9	C13—C8—C7	122.2 (3)
C1—C2—C3	120.2 (3)	C9—C8—C7	119.3 (3)
C1—C2—H2B	119.9	O2—C9—C10	117.3 (3)
C3—C2—H2B	119.9	O2—C9—C8	122.5 (3)
C4—C3—C2	120.0 (3)	C10—C9—C8	120.2 (3)
C4—C3—H3A	120.0	C11—C10—C9	120.4 (3)
C2—C3—H3A	120.0	C11—C10—H10A	119.8
C5—C4—C3	120.1 (3)	C9—C10—H10A	119.8
C5—C4—H4A	120.0	C12—C11—C10	119.6 (3)
C3—C4—H4A	120.0	C12—C11—H11A	120.2
C4—C5—C6	120.2 (3)	C10—C11—H11A	120.2
C4—C5—H5A	119.9	C13—C12—C11	121.4 (3)
C6—C5—H5A	119.9	C13—C12—Br1	118.8 (2)
C5—C6—C1	119.2 (3)	C11—C12—Br1	119.8 (2)
C5—C6—C7	118.5 (3)	C12—C13—C8	119.8 (3)
C1—C6—C7	122.2 (3)	C12—C13—H13A	120.1
O1—C7—C8	121.6 (3)	C8—C13—H13A	120.1
			
C6—C1—C2—C3	−3.1 (5)	C6—C7—C8—C9	170.9 (2)
C1—C2—C3—C4	1.4 (5)	C13—C8—C9—O2	−175.9 (3)
C2—C3—C4—C5	1.4 (5)	C7—C8—C9—O2	0.6 (4)
C3—C4—C5—C6	−2.5 (5)	C13—C8—C9—C10	3.4 (4)
C4—C5—C6—C1	0.9 (4)	C7—C8—C9—C10	179.9 (3)
C4—C5—C6—C7	176.9 (3)	O2—C9—C10—C11	176.6 (3)
C2—C1—C6—C5	1.9 (4)	C8—C9—C10—C11	−2.8 (5)
C2—C1—C6—C7	−173.9 (3)	C9—C10—C11—C12	−0.5 (5)
C5—C6—C7—O1	−48.6 (4)	C10—C11—C12—C13	3.1 (5)
C1—C6—C7—O1	127.3 (3)	C10—C11—C12—Br1	−176.9 (2)
C5—C6—C7—C8	130.9 (3)	C11—C12—C13—C8	−2.4 (4)
C1—C6—C7—C8	−53.2 (4)	Br1—C12—C13—C8	177.60 (19)
O1—C7—C8—C13	166.7 (3)	C9—C8—C13—C12	−0.9 (4)
C6—C7—C8—C13	−12.8 (4)	C7—C8—C13—C12	−177.2 (2)
O1—C7—C8—C9	−9.6 (4)		

### Hydrogen-bond geometry (Å, °)

**Table:**

*D*—H···*A*	*D*—H	H···*A*	*D*···*A*	*D*—H···*A*
O2—H2A···O1	0.82	1.85	2.570 (3)	146
C13—H13A···O2^i^	0.93	2.59	3.475 (3)	160

Symmetry codes: (i) *x*, −*y*−1/2, *z*−1/2.
